# Aberrant Activity in Degenerated Retinas Revealed by Electrical Imaging

**DOI:** 10.3389/fncel.2016.00025

**Published:** 2016-02-08

**Authors:** Günther Zeck

**Affiliations:** Neurochip Research Group, Natural and Medical Sciences Institute at the University of TübingenReutlingen, Germany

**Keywords:** ganglion cells, rod-degeneration, microelectrode array, extracellular recording, mouse retina

## Abstract

In this review, I present and discuss the current understanding of aberrant electrical activity found in the ganglion cell layer (GCL) of rod-degenerated (*rd*) mouse retinas. The reported electrophysiological properties revealed by electrical imaging using high-density microelectrode arrays can be subdivided between spiking activity originating from retinal ganglion cells (RGCs) and local field potentials (LFPs) reflecting strong trans-membrane currents within the GCL. RGCs in *rd* retinas show increased and rhythmic spiking compared to age-matched wild-type retinas. Fundamental spiking frequencies range from 5 to 15 Hz in various mouse models. The rhythmic RGC spiking is driven by a presynaptic network comprising AII amacrine and bipolar cells. In the healthy retina this rhythm-generating circuit is inhibited by photoreceptor input. A unique physiological feature of *rd* retinas is rhythmic LFP manifested as spatially-restricted low-frequency (5–15 Hz) voltage changes. Their spatiotemporal characterization revealed propagation and correlation with RGC spiking. LFPs rely on gap-junctional coupling and are shaped by glycinergic and by GABAergic transmission. The aberrant RGC spiking and LFPs provide a simple readout of the functionality of the remaining retinal circuitry which can be used in the development of improved vision restoration strategies.

## Motivation: Electrical Imaging of a Retinal Cell Layer

Imaging the electrical activity in three-dimensional neuronal tissue such as the retina relies on sensors which monitor changes of the transmembrane potential or of intracellular ion concentrations. The generation of action potentials leads to changes of intracellular calcium, which are revealed by fluorescence imaging of calcium indicators. For an introduction to calcium imaging in the retina and a comparison with electrical recording see the literature (Briggman and Euler, 2011). Changes of the transmembrane potential and associated transmembrane currents during action potentials or sub-threshold membrane fluctuations modulate the extracellular potential, which is recorded by extracellular electrodes (Fromherz, 2002; Stett et al., 2003). High-frequency (~1 kHz) changes of the extracellular potentials are assigned to action potentials, while low-frequency changes (5–100 Hz) are assigned to field potentials (Buzsáki et al., 2012; Einevoll et al., 2013).

In retina research, the most studied retinal layer from a physiological perspective is the ganglion cell layer (GCL), due to its easy accessibility in *ex vivo* preparations. Throughout this review, I refer to simultaneous electrical recording by thousands of micrometer-sized electrodes with high spatial (~10–30 μm) and temporal resolution (~20 μs) as electrical imaging, in accordance with previous studies (Zeck et al., 2011; Greschner et al., 2014). The specific ganglion cell spike patterns generate one type of “electrical image” of the retinal ganglion cell layer. A second type of “electrical images” is obtained by investigating field potentials in the low frequency range. In the following sections, I will review our current knowledge of these two physiological properties (spiking and LFPs) in the GCL of different rod-degenerated (*rd*) mouse retinas.

## Electrical Imaging of Spiking Activity in the Ganglion Cell Layer

Among the first results revealed by electrical imaging of the GCL was the wave-like spiking activity (“retinal wave”) in the developing retina (Meister et al., 1991). This activity reflects near-synchronous ganglion cell activity over several millimeters, separated by second-long periods of inactivity. Recently, the development of high-density CMOS-based MEAs comprising several thousand electrodes densely packed in a few square millimeters (Eversmann et al., 2003; Heer et al., 2007; Berdondini et al., 2009) has enabled imaging of retinal waves and their propagation patterns with unprecedented spatial and temporal resolution (Maccione et al., 2014), revealing their shrinkage during ontogeny to the size of the spatial receptive fields of retinal ganglion cells (RGCs).

In the healthy and mature retinas, wave-like activity ceases under physiological conditions. Two important findings revealed by simultaneous electrical recording from multiple ganglion cells are relevant for the understanding of RGC activity in photoreceptor-degenerated retinas: (i) Analysis of pair-wise RGC activity demonstrated functional coupling driven by either reciprocal/gap-junctional coupling between RGCs, by common input from one presynaptic bipolar cell, or by a combination of both modalities (Brivanlou et al., 1998). Correlated activity decreases with developmental age (Demas et al., 2003) but is maintained throughout adulthood for certain cell types. The coupling strength is light dependent (Hu et al., 2010). Electrical coupling between OFF alpha-like RGCs but not between ON alpha-like RGCs has been reported (Hu et al., 2010). Electrical coupling synchronizes the activity of neighboring RGCs, while cells of different polarity (ON vs. OFF RGCs) show phase-shifted activity. (ii) Secondly, the disruption of photoreceptor input achieved by pharmacological intervention or by bleaching of the retina unmasked spontaneous rhythmic activity in RGCs, as revealed by calcium imaging (Toychiev et al., 2013b), patch clamp recording (Toychiev et al., 2013b) or by electrical imaging using CMOS-MEAs (Menzler et al., 2014). This result suggests that rhythmic RGC activity in the mature retina may be triggered by the missing photoreceptor input.

Indeed, in the photoreceptor-degenerated *rd1* retina, extracellular recordings from large populations of RGCs measured increased spontaneous activity as compared to the activity in age-matched wild-type retinas (Ye and Goo, 2007; Stasheff, 2008), revealing hyperactivity and for some cells rhythmic activity. The result of elevated and rhythmic ganglion cell spiking has been confirmed and extended by many others, including recordings with CMOS MEAs (Menzler and Zeck, 2011; Menzler et al., 2014) or patch clamp pipettes (Margolis et al., 2008; Borowska et al., 2011; Yee et al., 2012). Ganglion cell hyperactivity and rhythmicity has been reported in a second *rd* mouse strain (*rd10*; Goo et al., 2011; Stasheff et al., 2011; Biswas et al., 2014; Menzler et al., 2014) as well as in mouse models in which synaptic transmission between photoreceptors and bipolar cells is dysfunctional (Crx^−/−^ and *nob mouse*; Demas et al., 2006; Soto et al., 2012). The two measures, *hyperactivity* and *rhythmicity* are distinct quantities. Hyperactivity is evaluated as the mean number of spikes counted over a time period of several seconds. Rhythmicity is revealed as a peak in the power spectra of the transmembrane currents (Yee et al., 2012) or in the spike-train autocorrelograms (Stasheff, 2008; Menzler and Zeck, 2011). RGC hyperactivity has been detected in *rd1* and in *rd10* when compared to wild-type retinas recorded under similar experimental conditions. The absolute spike rate values differ between the studies, with lower spike rates when ACSF was used as a recording medium (Ye and Goo, 2007; Stasheff, 2008; Goo et al., 2011; Stasheff et al., 2011) and higher values with Ames’ medium (Menzler and Zeck, 2011; Margolis et al., 2014; Ivanova et al., 2015). The spiking rhythmicity reported in these studies exhibits a maximum in the range of 4–15 Hz in all studied retinas. This parameter appears to be independent of experimental conditions (recording buffer, temperature range) but varies between strains. The oscillatory ganglion cell activity found in *rd1* RGC exhibits the highest frequency (~10 Hz), while this value declines for *rd10* (5–7 Hz) or nob mice (4 Hz). However, the reported rhythmicity is not seen in all RGCs and appears to vary over time (Goo et al., 2011; Biswas et al., 2014). Rhythmic RGC activity therefore needs to be understood as an intrinsic property of photoreceptor-degenerated or of photoreceptor-deficient retinas (see explanation below) which occurs to different degrees across the GCL.

### Mechanism of Rhythm Generation and its Implications for RGC Activity

The cellular origin of the rhythmic ganglion cell activity has been reviewed in detail (Trenholm and Awatramani, 2015). Briefly, AII amacrine cells are thought to act as rhythm generators. They display rhythmic transmembrane voltage oscillations of ~10 Hz when isolated from synaptically coupled cells by pharmacological blockers (Choi et al., 2014; Margolis et al., 2014). An alternative explanation suggests the rhythm may be generated in the electrically-coupled network of ON bipolar—AII amacrine cell (Trenholm et al., 2012). In both models the interplay between fast activating sodium channels and slower potassium channels (M-channels) in hyperpolarized AII cells evokes the rhythm generation. Although a quantitative evaluation of the different oscillatory frequencies (4–15 Hz among strains, detailed in the previous section) is missing, the increased rhythmic frequency of AII transmembrane voltage for depolarized midpoint membrane potentials (Choi et al., 2014) indicates a pure biophysical mechanism. In addition to the AII amacrine—bipolar cell rhythm generator, a second rhythmic network in the outer retina which shows stronger degeneration has been reported and reviewed by Euler and Schubert (2015).

The rhythm generation by the AII amacrine—ON bipolar cell network predicts a phase-shifted activity in ON and OFF RGCs. OFF bipolar cells, which excite the OFF RGCs, receive glycinergic input from AII amacrine cells while ON bipolar cells, which excite ON RGCs, are connected to AII amacrine cells by gap junctions. Indeed, phase-shifted activity has been recorded in double-patch clamp experiments in *rd1* for nearby alpha-like cell pairs of opposite polarity whereas there was no phase shift between alpha-like RGCs of the same polarity (Margolis et al., 2008). However, random phase-shifted activity between nearby RGC pairs (separation <200 μm) has been inferred from spike-train cross-correlation analysis of unspecified cell types (Menzler et al., 2014). This result strongly suggests that additional neurons, synaptically connected to RGCs, are rhythmic in *rd1* retinas (Margolis et al., 2014). In contrast, in wild-type retinas, where rhythmic activity in RGCs was induced by suppressing the photoreceptor input (Menzler et al., 2014), the phase shifted spiking of nearby RGCs was either zero or in a relatively narrow range of 60–90°. A similar result was found for a small number of rhythmic r*d10* RGCs.

In conclusion, the phase-shifted RGC activity predicted by the current model of rhythm generation has been confirmed for alpha-like RGCs in *rd1* and for unspecified RGCs in *wt* and *rd10*. However, arbitrary phase shifted RGC activity in *rd1* indicates that additional neurons in these retinas are rhythmic. The implications of concerted rhythmic activity for a multi-cellular behavior will be discussed in the following section.

## Local Field Potentials in the Ganglion Cell Layer of Rod-Degenerated Retinas

The RGC spiking properties do not provide a complete description of the functional changes occurring in *rd* retinas. Patch clamp recordings reveal strong sub-threshold oscillations of transmembrane potential (Choi et al., 2014) with frequencies in the same range as the spiking activity. Voltage clamp recordings at different holding potentials demonstrated that the excitatory and inhibitory input currents are rhythmic—although to a different degree in different cell types (Margolis et al., 2008; Yee et al., 2014). These single cell properties translate at a global level to low-frequency field potentials as revealed by MEA recordings. The changes of the extracellular voltage caused by correlated transmembrane currents are recorded as local field potentials (LFPs), given that they are spatially restricted within millisecond-long time intervals. LFPs have been electrically imaged in various brain areas, such as the well-known propagation along the tri-synaptic hippocampal formation (Ferrea et al., 2012) or in cortical structures (Viventi et al., 2011) revealing epileptiform activities. In the healthy and pharmacologically unperturbed mammalian retina LFPs have never been reported. This is most probably attributed to the small local circuits projecting to different types of ganglion cell and to the time-balanced excitatory and inhibitory input. LFPs occur at a time scale of tens of milliseconds, whereas excitation and inhibition are balanced within a few milliseconds.

In rod-photoreceptor degenerated mouse retinas (*rd1* and *rd10*) low-frequency extracellular voltage changes have been reported by different labs (Ye and Goo, 2007; Goo et al., 2011; Menzler and Zeck, 2011; Biswas et al., 2014; Menzler et al., 2014). The LFP frequencies are of the same value as the rhythmic spiking observed in these retinas, i.e., 5–15 Hz. Moreover, LFP maxima and spiking often correlate without time lags (Menzler and Zeck, 2011). This finding together with the observation that LFPs exist without spiking RGCs was an early indicator that rhythmic presynaptic activity drives the aberrant RGC spiking.

**Figure 1:**
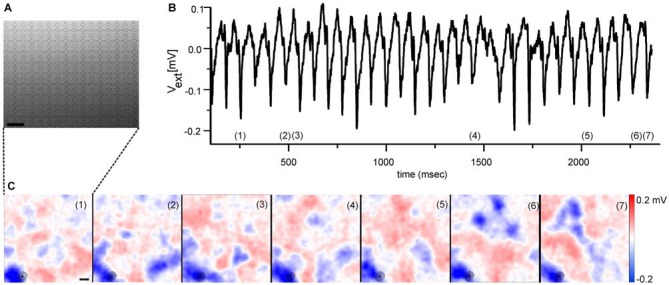
**Electrical imaging the rhythmic local field potentials (LFPs) in the ganglion cell layer (GCL) using high-density microelectrode arrays. (A)** Raster electron micrograph displaying part of a high-density microelectrode array. Round structures represent the recording electrodes. Scale bar: 32 μm. Figure modified from Bertotti et al. (2014). **(B)** Extracellular voltage trace recorded by one selected electrode [marked with circle in the lower left corner of each image in **(C)**] reveals the rhythmically occurring LFP. The voltage trace has been low-pass filtered (<100 Hz). **(C)** Selected color coded images of the extracellular voltage recorded by a high-density microelectrode array as shown in **(A)**. The numbers in each image correspond to numbers in **(B)**, which identify the time of maximal LFP amplitude. The periodically occurring LFPs share a high spatial similarity. Scale bar: 100 μm. **(B,C)** modified from Menzler and Zeck (2011).

A quantitative analysis of the detected LFP size is inherently difficult, as they develop over time in size and amplitude. However, two qualitative LFP features are worth noting. Their size at maximal amplitude extends over an area of ~200 μm (see Figure 1 and Menzler and Zeck, 2011) suggesting that only a few cells contribute to the initiation of the rhythm. Secondly, LFPs show mostly negative extracellular voltages (blue colors in Figure 1C), indicating strong inward currents. The outward currents are spatially more widely distributed, reflected in the shallower red colors seen in Figure 1C. Similar findings can be inferred from the studies by Menzler and Zeck (2011), Biswas et al. (2014) and correspond to the strong inward currents reported in Yee et al. (2012). A quantitative evaluation which relates the LFPs through a current source density analysis to the generating cells and cellular compartments (Ness et al., 2015) has not been performed so far.

As for the spiking properties, spatial electrical imaging the LFPs reveals qualitative differences between *rd1* and *rd10* retinas. In *rd1* retinas the “pulsatile” LFPs propagate at a median speed of 8 mm/sec (Menzler and Zeck, 2011). In the *rd10* GCL it is unclear if LFP propagation occurs (Menzler et al., 2014). The putative propagation reported in a previous study by Biswas et al. (2014) is somewhat difficult to interpret given the large spacing between electrodes. Propagation was suggested to depend on electrical coupling; however there is no conclusive experimental evidence transforming propagating LFPs into stationary ones. Furthermore, the repetitive occurrence of LFPs in the same retinal portion (see Figure 1) and evidence provided by Menzler and Zeck (2011), indicates a considerable spatial variability and LFP dynamics which requires a thorough quantitative analysis in future studies.

**Figure 2:**
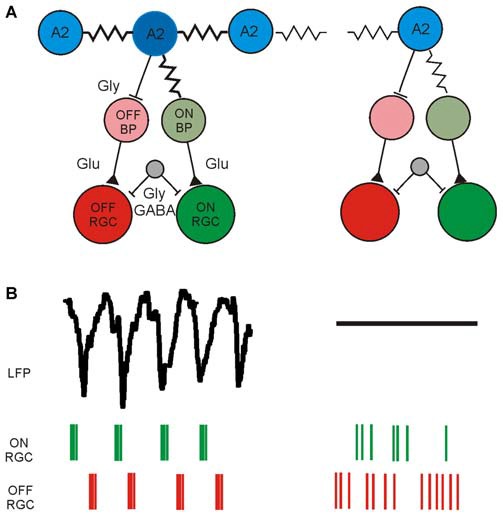
**Schematic diagram of synaptically connected retinal neurons generating rhythmic activity in the GCL of photoreceptor-degenerated retinas in the low and high frequency range. (A)** Simplified schematic synaptic connectivity in *rd* retinas. The rhythmic activity is generated and spreads in the electrically coupled network of AII amacrine cells and ON cone-bipolar cells (ON CB) and propagates through glutamatergic excitation to ON-type RGCs. Through glycinergic inhibition the rhythmic activity reaches OFF RGCs. An unidentified cell (gray) modulates the rhythmic spiking through GABAergic and/or glycinergic input. The rhythmic activity does not occur over the entire retina, symbolized in the weakly coupled network on the right. Part of the figure modified from (Margolis et al., 2014). **(B)** Low-frequency LFP and rhythmic, high-frequency spiking in two different retinal ganglion cell types (ON RGC and OFF RGC) driven by the rhythmic presynaptic network. Rhythmic LFPs and RGC spiking is probably recorded only across strongly coupled areas of the RGC layer, whereas in weakly coupled regions the LFP size decreases and the spiking becomes arrhythmic.

The current knowledge about the retinal neurons involved in the generation of rhythmic activity and the corresponding functional readout using electrical imaging is summarized in Figure 2. This functional characterization is based on available methodology; including appropriate filtering (low-pass and high-pass), spike and LFP identification, spike sorting, computation of auto- and cross-correlograms and calculation of power spectral densities. In addition to the aberrant, rhythmic activity detected in photoreceptor-degenerated retinas one should remember that a considerable percentage of RGCs show normal activity, most probably in areas of the GCL with less circuit degeneration.

In the following last section, I discuss how aberrant ganglion cell activity and the spatio-temporal LFP dynamics affect strategies for visual restoration in *rd* retinas.

## Advances and Current Limitations of Visual Restoration in Rod-Degenerated Retinas

A prerequisite for visual restoration in patients who are blind due to loss of photoreceptors is the survival of inner retinal neurons and their synaptic connections to higher visual areas. In *rd* mouse models previous work has revealed changes of bipolar cell morphology (Strettoi et al., 2002; Gargini et al., 2007; Barhoum et al., 2008) but a well preserved RGC morphology (Mazzoni et al., 2008) and mosaic-like arrangement of at least two RGC types (Lin and Peng, 2013). Furthermore, RGCs in *rd1* and in *rd10* retina retain their synaptic connections with higher visual centers (Bi et al., 2006; Lagali et al., 2008; Lin et al., 2008; Ivanova et al., 2015). Specifically, the aberrant RGC activity propagates to higher brain areas and may hinder visual coding (Ivanova et al., 2015).

Strategies for restoration of vision may therefore address the following questions, assuming that the lessons learned in blind mice translate to the human case. How can the aberrant ganglion cell activity be overcome by external stimuli and to what degree do external stimuli evoke near-physiological RGC response patterns?

The first question has been tackled by employing pharmacological intervention (Toychiev et al., 2013a; Barrett et al., 2015). For a certain number of RGCs and a relatively early degeneration age the application of gap junction blockers reduced hyperactivity and at the same time enabled RGC activation either by electrical stimulation (Toychiev et al., 2013a) or by optogenetic cell transfection followed by light stimulation (Barrett et al., 2015). While the reduction of gap-junctional coupling might be beneficial for approaches targeting the RGCs directly, such as epiretinal electric stimulation (Chuang et al., 2014) or optogenetic activation of ChR2 expressing RGCs (Bi et al., 2006), it might corrupt visual processing in strategies targeting the inner retinal circuitry, such as subretinal electrical stimulation (Zrenner et al., 2011) or optogenetic intervention at the bipolar cell level (Lagali et al., 2008; van Wyk et al., 2015). Although MEAs have been used to detect evoked RGC activity (Barrett et al., 2015), no electrical imaging of the GCL has been performed.

The second question of how close the artificially-evoked responses in blind retinas resemble light-activated activity in healthy retinas has been addressed for electrical (O’Hearn et al., 2006; Ryu et al., 2010; Goetz et al., 2015; Lorach et al., 2015) and for optogenetic stimulation (Busskamp et al., 2010; van Wyk et al., 2015). Subretinal electric stimulation of dystrophic rat retina evoked spatially selective RGC response patterns which were of the same size as light-evoked receptive fields in healthy retinas (Lorach et al., 2015). This remarkable result was achieved by recording the evoked RGC response using a high-density MEA. However, the stimulation method (spatial white noise) used to map the electrically evoked receptive fields did not reveal putative rhythmic RGC activity, as reported in another study (Ryu et al., 2010) for pulsatile stimulation of *rd1* RGCs. Common to the electrical stimulation approaches in blind retinas is the very small percentage of activated OFF RGC responses (Goetz et al., 2015). Therefore light activation of optogenetically transduced halorhodopsin in remaining cones (Busskamp et al., 2010) or activation of a chimeric melanopsin-mGluR6 molecule in rd1 ON bipolar cells (van Wyk et al., 2015) is attractive, as it restored ON and OFF RGC response polarities in recorded RGCs. For optogenetic stimulation little is known so far about the distortion by aberrant RGC or LFP activity, since the above-cited studies relied on single cell recordings.

In future, electrical imaging of the retinal GCL may improve future vision strategies in two ways. First, it can indicate if vision restoration is successful by comparing the artificially induced RGC patterns to physiological patterns. Several of the above cited studies performed a basic characterization of the induced activity, which can easily be extended to a comprehensive electrophysiological picture. Second, electrical imaging and analysis of the RGC population activity in blind retinas may reveal to what degree the aberrant rhythmicity impairs the coding of artificially evoked responses. Studies in frog and in cat retinas indicate a beneficial effect of oscillatory retinal activity for visual coding (Ishikane et al., 2005; Koepsell et al., 2009) while a recent study using blind retina underlines the distortion introduced by the aberrant activity (Ivanova et al., 2015).

## Author Contributions

GZ wrote the manuscript.

## Funding

Funding of this work was in part obtained from the German Federal Ministry of Education and Research through grant no. 1312038.

## Conflict of Interest Statement

The author declares that the research was conducted in the absence of any commercial or financial relationships that could be construed as a potential conflict of interest.
